# Tomographic and Tension Analysis of Polypropylene Reinforced with Carbon Fiber Fabric by Injection Molding

**DOI:** 10.3390/ma16186231

**Published:** 2023-09-15

**Authors:** Michal Wieczorowski, Alejandro Pereira, Teresa Prado, Alberto Lopez-Blanco, Karol Grochalski, Wieslaw Grabon, M. Consuelo Perez

**Affiliations:** 1 Faculty of Mechanical Engineering, Poznan University of Technology, Piotrowo Street 3, 60-965 Poznan, Poland; karol.grochalski@put.poznan.pl; 2 Manufacturing Engineering Group (GEF) EEI Campus Lagoas, Universidade de Vigo, 36310 Vigo, Spain; apereira@uvigo.es (A.P.); tprado@uvigo.es (T.P.); albertolopezb@alumnado.uvigo.gal (A.L.-B.); 3 Faculty of Mechanical Engineering and Aeronautics, Rzeszow University of Technology, Powstancow Warszawy 8 Street, 35-959 Rzeszow, Poland; wgrabon@prz.edu.pl; 4 ENCOMAT, Centro de Investigación en Tecnologías, Energía y Procesos Industriales, Universidade de Vigo, 36310 Vigo, Spain; mcperez@uvigo.gal

**Keywords:** PP, CF reinforcement, over-injection molding, composites

## Abstract

The use of thermoplastic materials has had significant growth in recent years. However, with great mechanical requirements, thermoplastics have limitations to their use. To improve these restrictions, these materials are reinforced to obtain better properties. Polypropylene is one of the most versatile polymers and is used in almost all modern industries. Thus, the aim of this study is to create composite materials that offer performance for various industrial fields using carbon fiber fabric reinforcement, which is an inexpensive material widely used by the aerospace, automotive, and marine industries. The samples are produced by the over-injection molding of polypropylene. The investigation is focused on the impact of two critical control parameters in the injection molding process: temperature and pressure. Twelve experiments have therefore been considered, taking into account the combination of three factors: the presence or absence of carbon fiber fabric reinforcement, three levels of temperature (200 °C, 220 °C, and 240 °C), and two injection pressures (5000 kPa and 10,000 kPa). To evaluate the influence of these factors, three analyses were carried out: first, on the samples’ shrinkage using a portable metrology-grade 3D laser scanner; second, on the internal defects using computed tomography (CT); and third, on the mechanical properties with tensile tests. From the results obtained, it is observed that the mold shrinkage fell slightly when PP samples were reinforced with carbon fiber, with both materials (PP and carbon-fiber-reinforced PP) having linear behavior with temperature. It is also noticed that polypropylene behaves as a crystalline material when processed at higher temperatures and pressures. From tests on the mechanical properties, it is concluded that the mean yield strength of PP-CF for injection temperatures of 220 °C and 240 °C represents an increase of 43% compared to the non-reinforced material.

## 1. Introduction

Thermoplastics are a group of materials composed of polymers and held together by intermolecular forces, resulting in linear or branched structures. When exposed to high temperatures, these materials become flexible and deformable, allowing them to be melted and reshaped multiple times. The demand for thermoplastic materials has experienced significant growth in recent years, driven by their expanding range of applications [1,2]. They are being utilized in the development of new products as well as in the substitution of traditional materials, such as metals. The combination of their affordability, excellent thermal and mechanical properties, and low specific weight has played a major role in their widespread adoption.

When it comes to designing thermoplastic composites, several crucial factors need to be taken into consideration. These composites consist of a thermoplastic matrix or binder combined with an immiscible reinforcement that is closely bound to the binder. The properties of the composite are predominantly influenced by the matrix, reinforcement, and adhesion between them. Mechanical properties, in particular, are greatly impacted by the choice of reinforcement. On the other hand, the matrix plays a vital role in determining other properties, including thermal behavior, durability, chemical resistance, and fire resistance. Ultimately, the adhesion between the reinforcement and matrix is essential for achieving the desired final properties [3].

The development of polypropylene and similar plastics has made it feasible to create personal computers and handheld calculators. These plastic materials possess outstanding characteristics such as a high heat distortion temperature, exceptional rigidity, effective electrical insulation, remarkable resistance to bending, and effortless molding capabilities [4]. 

Glassfiber-reinforced polymer composites dominate the polymer fiber composites industry, accounting for approximately 90% of its usage [5]. These composites incorporate glass fibers in various forms, including roving, mats, fabrics, and chopped fibers. They find extensive application in the production of boat hulls, yachts, tanks, bathtubs, roof gutters, pipes, and machine housings [6].

In the present work, the authors are interested in the application of carbon fiber fabric as a reinforcement material in polymers manufactured via over-injection molding. Previous work has already worked with over-injection-molded polyamide fabric with glass fiber reinforcement [7]. Carbon fiber fabric with high tensile toughness is extensively employed to reinforce polymer matrix composites for mechanical enhancement purposes. Carbon fibers have remarkable mechanical properties; however, they are widely recognized to exhibit inadequate wettability and adhesion to polymers due to their chemically inert and smooth surface. Chukov et al. [8] suggest thermoplastic-based composite materials reinforced with carbon fibers. They studied composites based on polysulfone reinforced with carbon fabrics using polymer solvent impregnation. This difficult adhesion of the woven carbon fibers makes the industrial process of continuous overmolding difficult. In fact, there is a lack of research on processing via the over-injection of carbon fiber fabric onto polypropylene [9,10]. Another novelty taken into account in this work has been the use of the computed tomography technique to examine the porosity of the composite material and to analyze the adhesion of the fibers to the matrix, taking into account the injection parameters.

The authors’ interest lies in the wide and cheap use of reinforced carbon fiber fabric in the aerospace, automotive, and marine industries, as well as in wind turbines and in the medical field, by injection mold, which is a technique used for the mass production of plastic parts. Quality consistency is essential in the injection molding process for maximizing the yield rate and minimizing the production cost, and this issue is more concerned with proceeding with recycled material. Chen et al. [11] propose an approach to the monitoring, prediction, and control of injection molding quality based on the clamping force increment characteristic, as determined by the measured tie-bar elongation. Zhao et al. [12] study the in situ ultrasonic measurement of molten polymers during the injection process. Their experiments were carried out to measure the melting temperatures of low-density polyethylene at different injection speeds and the melting temperatures at which measurement errors were less than 7.5%. 

The influence of two control parameters of the injection process is observed in the current work: temperature and pressure. According to numerous studies concerning the optimization of PP injection molding, the temperature and injection pressure have the greatest weight and influence on the mechanical properties and process improvement [13,14,15,16]. Seeger et al. [17] studied the melting point under high pressure. They concluded that the properties of the polymers (vinyl acetate content, melt flow index, molecular weight, isotactic index, crystallinity, density, and frequency of branching) are correlated with the change in the melting point with pressure (dTm/dp). 

Overall, the main objective and novelty of this research is to advance the computed tomography analysis and application of carbon-fiber-fabric-reinforced polypropylene manufactured through over-injection molding, with the ultimate goal of developing cost-effective composite materials for various industrial sectors. The dimensions of the samples were evaluated via laser scanning, which consisted of examining the variations between different samples with and without reinforcement. Another novelty in this work is the application of computed tomography (CT) [18] in order to evaluate material properties (porosity), which is a non-destructive technique in contrast with destructive tensile tests. 

## 2. Materials and Methods

**Figure 1:**
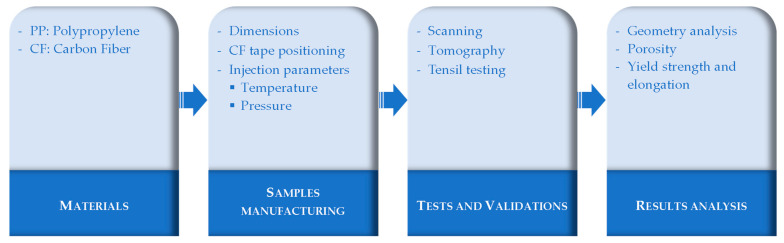
Experimental procedure.

The aim of the current work is to investigate improvements in the mechanical properties of parts made of polypropylene (PP). For this purpose, commercial carbon fiber tape has been used as a reinforcement in a base of PP. The methodology developed for the consecution of the reinforced composite material is based on the phases shown in Figure 1. 

### 2.1. Materials Selection

Injection molding is one of the most common manufacturing processes for polymer products. During the injection molding process, a polymer melt is forced into a cavity under high pressure, and the geometry of the cavity determines the shape of the final product. The mechanical properties of injection-molded articles are markedly affected by the temperature and velocity fields that the melted elements in the cavity are subjected to during the molding cycle [19].

The matrix material is a homopolymer used for general purpose injection molding applications. PP in pellet form was obtained from Lyondellbasell company, with the commercial name Moplen HP500N and the properties shown in Table 1 [20].

This polypropylene is reinforced with a carbon fiber material. This fabric is a 3 K 2 × 2 twill weave type, with polyamide fixing thread and a surface weight of 200 g/m^2^. The yarn type is TR30S.3K (3000 filaments per strand of yarn). The fabric does not unravel easily and can be cut into complex shapes without separating the warp and weft. It is ideal for combining with any type of thermosetting resin. It was obtained from Castro composite S.L. Table 2 [25] presents the specifications of the carbon fiber reinforcement. The amount of carbon fiber fabric material contributed to each specimen is 10 mm × 70 mm tissue slice, weighing 0.14 g and with a volume of 77 mm^3^. The cavity volume is 3892 mm^3^. The volume of PP injected is 3815 mm^3^. 

### 2.2. Manufacturing of Composite Samples

#### 2.2.1. Cavity Insert Design

Composite samples were produced in an ENGEL VICTORY 28-ton (ENGEL AUSTRIA GmbH, Schwertberg, Austria) injection molding machine with a 25 mm diameter screw. It is a two-plate injection mold machine equipped with interchangeable cavity inserts on the plate. The machined cavity and core are mounted onto the injection molding machine. 

**Figure 2:**
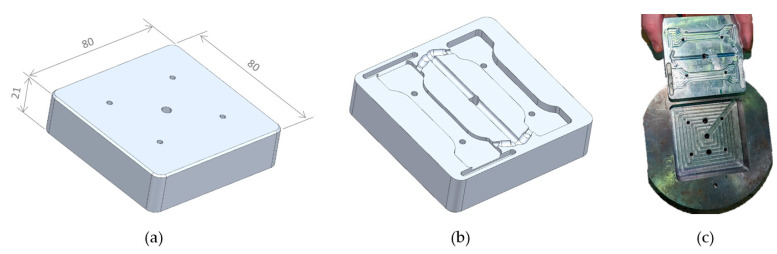
(**a**) Blank mold insert; (**b**) finished mold insert; (**c**) aluminum cavity insert and the mold base.

To take advantage of the base of the mold installed in the injection machine, the mold insert size selected was 80 × 80 × 21 mm. Aluminum 5083 was chosen for this study because it does not involve long production runs [26]. Figure 2 depicts the aluminum mold insert and the mold base, specific to this project. 

The authors investigated how to squirt the polymer melt into the cavity to guarantee the position and non-deformation of the carbon fiber tape, as well as how to obtain the correct adhesion of the carbon fiber fabric in the PP matrix in a different study. 

**Figure 3:**
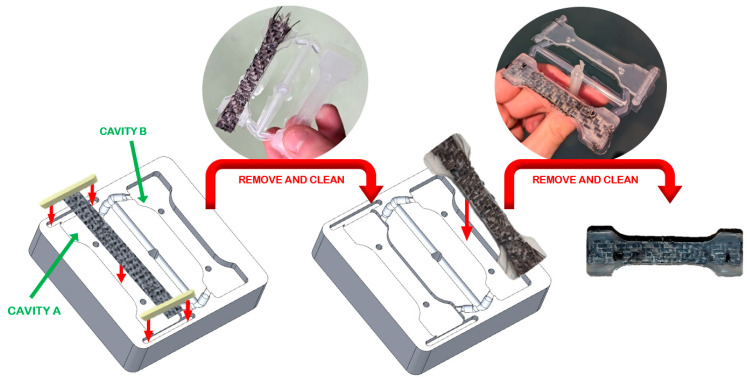
Injection steps of composite samples.

It was decided to obtain the samples in two injection steps, each sample being composed of two halves. In the first step, the fabric was placed and fixed by two inserts in the cavity A. After the injection, the half-sample was separated from runners and sprues, cleaned, and introduced in cavity B for the second injection step (Figure 3).

To avoid the fiber shifting during the injection process due to the impact on the fabric edge of a very high flow rate from the gate, the depth of runners in the gates was decreased. 

#### 2.2.2. Sample Dimensions 

**Figure 4:**
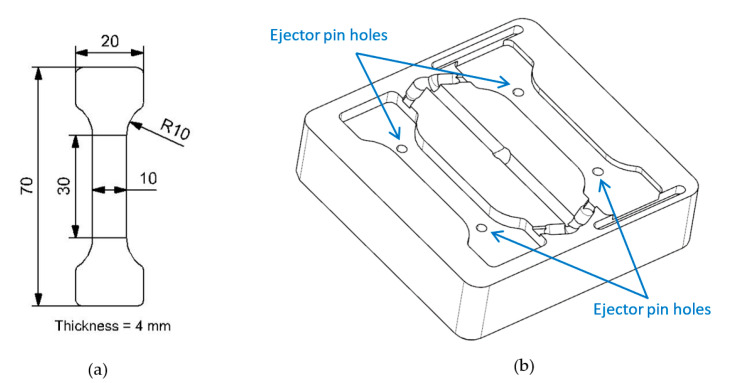
(**a**) Sample dimensions. (**b**) Position of cavities with respect to ejector pins.

The dimensions of the samples do not follow any standard because of the difficulty of adjusting the two half-sample cavities to remove them properly. Therefore, the two cavities were positioned in the mold insert, taking into account the position of the ejector pins. Two ejector pins were positioned over each cavity of the samples, as shown in Figure 4. 

#### 2.2.3. Injection Process

Two important injection molding parameters, injection temperature and pressure, were selected as the variable processing parameters. Injection temperature was set at 200, 220, and 240 °C. The injection pressure was set at two levels: 5000 and 10,000 kPa. The other processing parameters were kept constant, including a clamping force of 200 kN. The cooling time and the post-pressure time were set to 30 s and 5 s, respectively.

To analyze the improvement in the properties of the PP with the carbon fiber reinforcement, samples without reinforcement were also injected. One sample and five replicas were injected under the same conditions. In total, seventy-two samples were injected, considering the different combinations of reinforcement material, temperature, and pressure, corresponding to the designs of the experiments shown in Table 3. The code of each sample is composed of two numbers separated by dots: the first one is the combination of temperature and pressure (6 different combinations with 5000–10,000 kPa and 200–220–240 °C), and the second one is the number of the sample with that parameter’s combination; 0 is the sample, and from 1 to 5 are the replicas. These two numbers are preceded by a prefix: PP for samples without reinforcement and PP-CF those with the carbon fiber reinforcement. 

**Figure 5:**
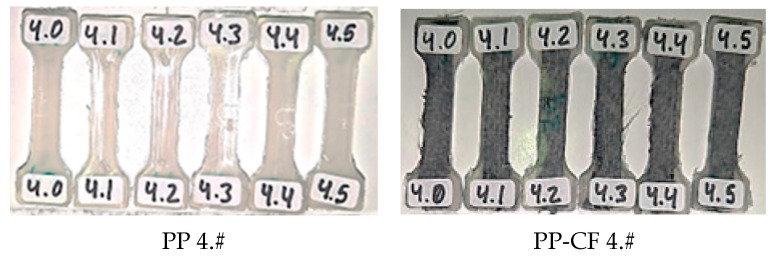
Samples of experiments 7 (PP 4.#) and 8 (experiments PP-CF 4.#), respectively.

In Figure 5, an example of the codes is shown for the samples injected at 220 °C and 50 bar of pressure, where the six samples of the original PP are labelled with PP 4.# (experiment 7) and the six samples reinforced with carbon fiber are labelled with PP-CF 4.# (experiment 8). 

### 2.3. Validation and Tests

#### 2.3.1. Metrological Controls

Once the composites were manufactured and before realizing the tests to evaluate their properties, the dimensions of the samples were evaluated to analyze if the fabric reinforcement has any influence during the contraction processes on said dimensions. The samples were measured using a portable metrology-grade 3D laser scanner, the Handy SCAN 3D BLACK Series, with an accuracy of 0.025 mm. 

**Figure 6:**
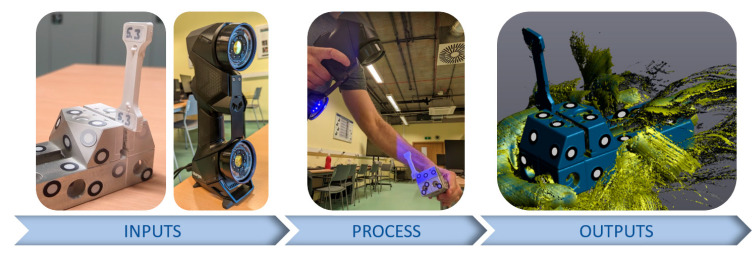
Scanning of samples using the Handy SCAN 3D BLACK Series.

To acquire the point clouds, an anti-reflection coating was applied to samples, and then the scanning process was carried out while holding the sample by hand and rotating it to obtain its full volume, as shown in Figure 6.

**Figure 7:**
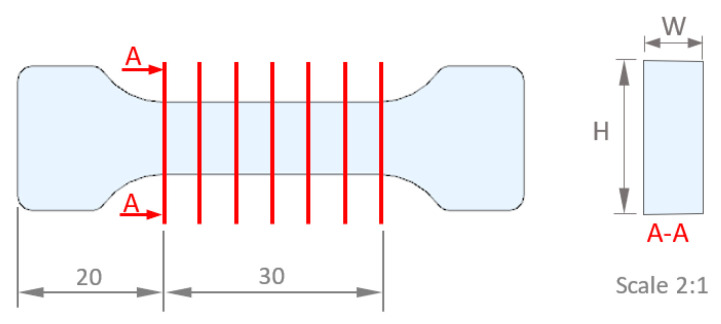
Sections for measurements.

After the point clouds were scanned, they were cleaned and converted to triangular mesh models (STL). The dimensions evaluated were the height and width in seven sections on the testing area, measuring both the dimensions for each section in the CAD file of each sample, as shown in Figure 7. Mean and standard deviation for width and height were calculated for each sample to analyze the geometry. 

#### 2.3.2. Internal Defects Inspection

Afterwards, the non-deformation of the carbon fiber tape during the injection process was evaluated, as were possible defects that appeared in the process, such as porosity. This analysis was realized by an X-ray Computed Tomography (CT) system, the Phoenix V|tome|x S240, scanning the same test area analyzed in the previous dimensional measurement. A computed tomography (CT) is a technique that is becoming increasingly popular for geometric measurements, including even surface topography [27]. It is useful for measuring composites with complex shapes and defined internal structures, which are often manufactured using additive techniques [28], with particular interest in metal additively manufactured parts [29]. It is a non-destructive test that allows for a volumetric evaluation of shapes and porosity, not only in terms of percentage but also in terms of pore location and size [30].

**Figure 8:**
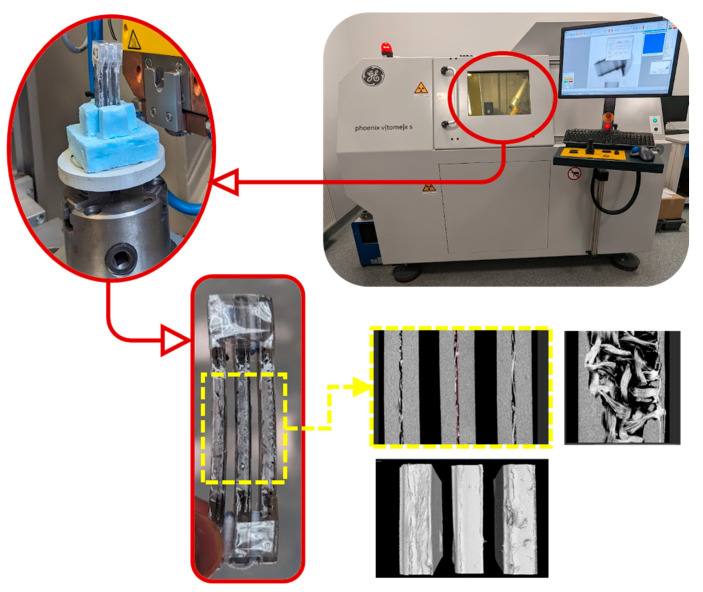
Computed Tomography scan of a set of three samples.

To optimize the time and cost of the CT testing, samples were grouped in sets of three to perform the scan, and subsequently, the group file was split into three files containing each sample of the group (Figure 8).

**Figure 9:**
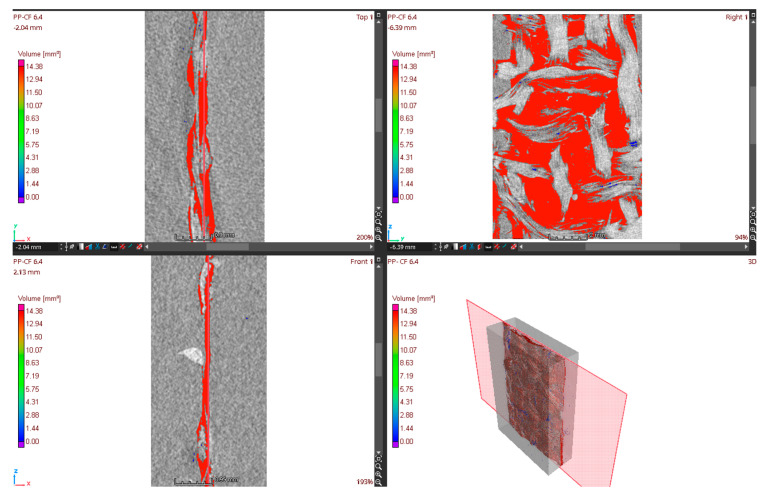
Porosity analysis in the sample PP-CF 6.2.

An analysis of porosity was carried out for each experiment. From the CT scan, besides the images of internal samples, the volume of the study area, the volume of pores per mm^3^, and the percentage of pores within the study area were also obtained (Figure 9). This percentage was the data used for the analysis.

#### 2.3.3. Mechanical Properties Testing

Tensile tests were performed on the samples to verify and compare the changes in the material properties with the porosity results obtained through CT due to the carbon fiber reinforcement and variation in the process conditions. 

**Figure 10:**
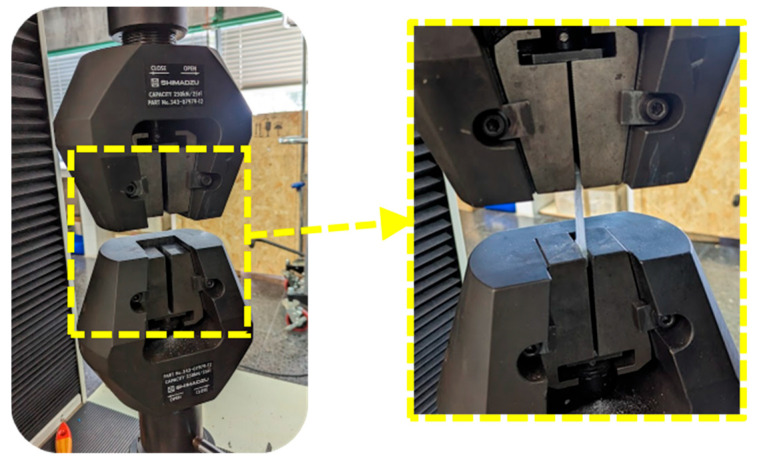
A view of tensile tests.

The tensile tests were performed 2 times for each experiment, i.e., a total of 24 measurements on a SHIMADZU model AG-I 250 Kn universal testing machine (Shimadzu corporation, Tokyo, Japan, Figure 10). The software used was TRAPEZIUM X (from Shimadzu), which has allowed the test parameters to be established as well as the test results in Excel format to be obtained for subsequent analysis.

The test parameters configured were as follows:

- a rate of 5 mm/s test speed;
- a maximum applied force of 2.5 kN;
- 10 mm × 4 mm × 16 mm as the calibrated volume.

Tensile test graphs were obtained and the yield strength and strain were evaluated to the subsequent analysis of mechanical properties. 

## 3. Results and Discussion

After the methodology and the execution of the experimental work, the data from the different tests were analyzed. In each experiment, two replicas were randomly selected for the tensile test, and with the results obtained for these replicas in the different tests carried out, the data shown in Table 4 were obtained.

### 3.1. Geometrical Results

**Figure 11:**
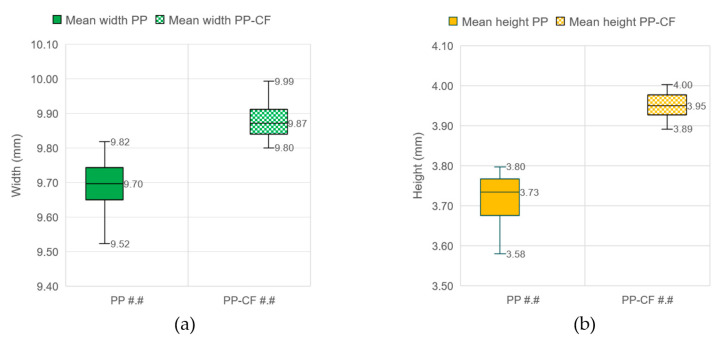
Comparison of dimensions after injection between PP samples without (PP #.#) and with (PP-CF #.#) carbon fiber reinforcement. (**a**) Mean width. (**b**) Mean height.

The box-and-whiskers plots shown in Figure 11 represent the behavior of the shrinkage after injection. The width and height in seven sections of each sample were calculated after scanning the samples with the laser scan, as described in the methodology. The mean of each dimension (width and height) was calculated for each sample. All sample measurements were taken two months after the injection process. Figure 11a illustrates the mean width of the 36 PP samples compared to the other 36 carbon-fiber-reinforced PP samples. Similarly, Figure 11b depicts a comparison of the mean height for samples with and without reinforcement. 

It can be seen that the mold shrinkage fell slightly when PP samples were reinforced with carbon fiber; the mold shrinkage rate was about 1.3% (0.13 mm width and 0.05 mm height). The PP samples without reinforcement had a higher mold shrinkage rate of 3% in width (0.30 mm) and 6.6% in height (0.27 mm). It can be stated that the reinforcement had an influence on the mold shrinkage of the PP material. The values of the shrinkage of the material without reinforcement were obtained from the bibliography [31,32,33], which gave experimental shrinkage of the PP from 1.8% to 4.8%, depending on the direction of the gate and injection process conditions. Uzman Jan et al. [34] studied the influence of the injection parameters on the shrinkage of polypropylene, obtaining, under the other conditions, an optimum temperature of 238° for reducing the shrinkage of the PP material. According to Kosciuszko et al. [35], it should be emphasized that the geometry of the injection moldings made of semicrystalline materials, whose glass transition temperature is lower than the working temperature, is not stable after removing them from the injection mold and cooling to the ambient temperature. 

**Figure 12:**
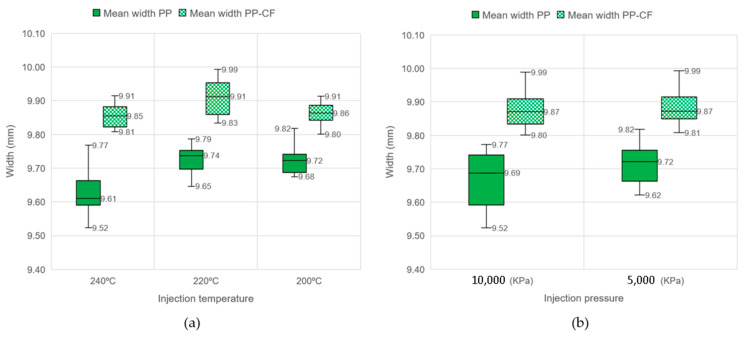
Comparison of width dimension at different injection parameters of PP samples without and with carbon fiber reinforcement. (**a**) At different temperatures. (**b**) At different pressures.

The effect of the injection molding parameters, temperature and pressure, on the final dimensions of the samples is presented in Figure 12. The graphs again demonstrate the lower shrinkage of the reinforced material for all the temperatures and pressures tested. This figure also reveals that there is no significant difference in the shrinkage of the PP-CF material at the evaluated parameters, of the order of 1.5% at 240 °C and 200 °C and 1.3% at both tested pressures. 

Taking a closer look at Figure 12a, it can be observed that both materials (PP and PP-CF) have a linear behavior with temperature, with the lowest mold shrinkage at 220 °C (0.9% for PP-CF and 2.6% for PP). It is worth noting that as the injection temperature decreases, the difference between the shrinkage of PP with and without reinforcement also decreases. A similar behavior is noticed for the heights of the sections of the samples. 

Likewise, Figure 12b shows the effect of the injection pressure on the width dimension. However, there seems to be a more significant influence of pressure on mold shrinkage in the case of the material without reinforcement, with a rate of 3.1% at 1000 kPa and 2.8% at 5000 kPa. These results are in agreement with Ryu et al. [36], who investigated the effect of the reinforcing factors on minimizing the shrinkage of PP composites. They concluded that the optimum condition for minimizing the directional shrinkage was the incorporation of 20 wt.% GF. 

### 3.2. Compute Tomography Results 

**Figure 13:**
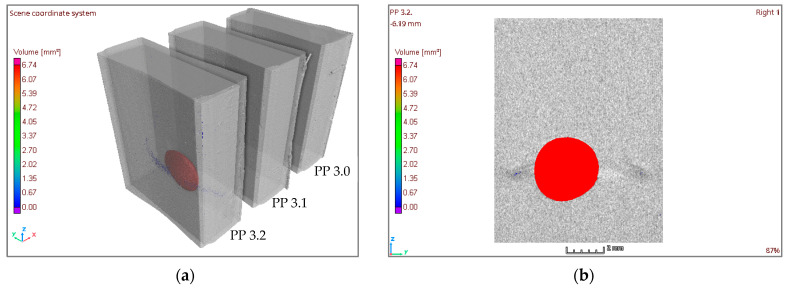
Samples of CT measurements of PP 3.X. (**a**) 3D image of samples PP 3.2, PP 3.1, and PP 3.0. (**b**) 2D image of sample PP 3.2.

The results obtained from the CT scan were gathered, and the volume of the study area, the volume of pores per mm^3^, and percentage of pores within the study area were obtained. Figure 13a presents a 3D image of the three samples (PP 3.2, PP 3.1, and PP 3.0) scanned together, and Figure 13b shows the details of the pore in the PP 3.2 sample, which has a diameter of about 2.5 mm. The number of samples rejected for non-conformity due to a high degree of porosity was insignificant. The sample in this figure was one of the few samples rejected because of the large pore size present. Most of the samples had no pores or had a percentage under 0.2%. 

Regarding the volume of pores from the CT measurements, by looking at Table 4, it can be noted that practically all the replicas without reinforcement selected have no porosity. These few samples, such as PP 3.2 mentioned above, were rejected, and therefore, an analysis of these samples is not required.

**Figure 14:**
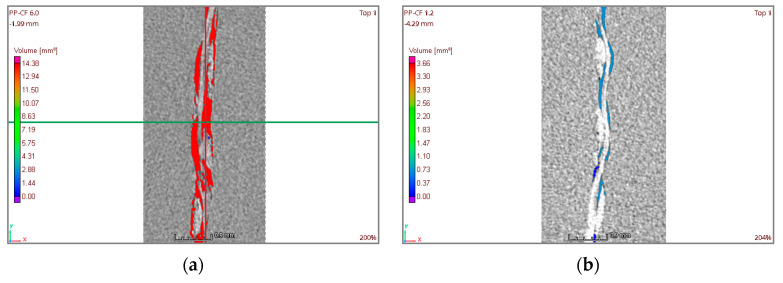
Samples PP-CF. (**a**) 2D image of sample PP-CF 6.0. (**b**) 2D image of sample PP-CF 1.2.

CT images were also used to check that the carbon fiber fabric does not shift in the sample during the injection process and that it remains in the central position. The CT results confirm that the carbon fiber remains in the correct position and it is centered, as illustrated in Figure 14 [37]. 

**Figure 15:**
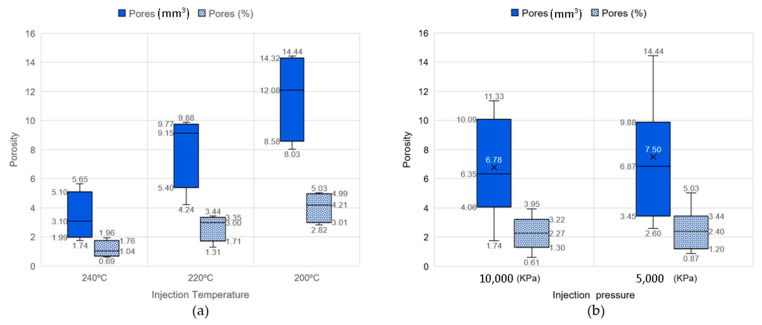
Comparison of porosity at different injection parameters. (**a**) At different temperatures. (**b**) At different pressures.

Figure 14a corresponds to the PP-CF 6.0 sample with the highest percentage of porosity (14.4 mm^3^, 5%), and Figure 14b shows the PP-CF 1.2 sample with the lowest porosity (1.74 mm^3^, 0.61%). When relating the porosity of these two samples to the injection parameters (PP-CF 6.0 injected at 200 °C and 5000 kPa and PP-CF 1.2 injected at 240 °C and 10,000 kPa), the results suggest a relationship between the injection parameters and porosity, which is analyzed in Figure 15, where the porosity is presented versus the injection parameters, temperature, and pressure. 

As for the injection temperature, Figure 15a denotes an important correlation between temperature and porosity. It could be concluded that the higher the temperature, the lower is the porosity. Regarding the injection pressure, Figure 15b presents a slight correlation between the pressure and the porosity. The porosity exhibits an inverse relationship with pressure, leading to a decrease in the mean porosity from 7.50 mm^3^ to 6.78 mm^3^ as the pressure increases from 5000 kPa to 10,000 kPa. 

**Figure 16:**
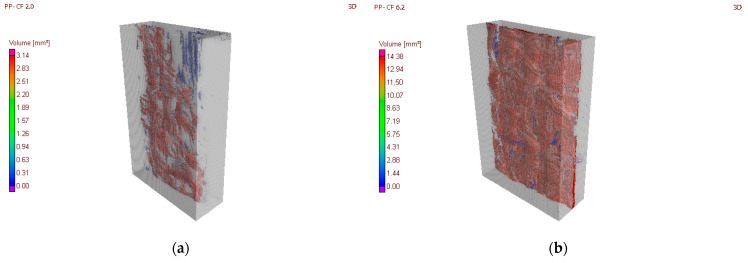

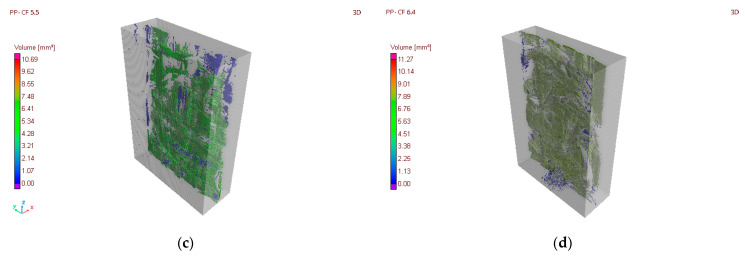
Pores volume analysis. (**a**) Sample PP-CF 2.0. (**b**) Sample PP-CF 6.2. (**c**) Sample PP-CF 5.5. (**d**) Sample PP-CF 6.4.

The relationship between porosity and the injection parameters is observed in the CT images in Figure 16. Regarding temperature, Figure 16a,b illustrate the significant influence of temperature on the porosity at the same pressure (5000 kPa). The PP-CF 2.0 sample, injected at 240 °C, has a total porosity of 3.45 mm^3^, which is significantly lower than the porosity of the PP-CF 6.2 sample injected at 200 °C, with a porosity of 13.94 mm^3^. Figure 16c,d show the slight influence of the injection pressure on the porosity at the same temperature. The PP-CF 5.5 sample, injected at 10,000 kPa, has a total porosity of 6.54 mm^3^, which is significantly lower than the porosity of the PP-CF 6.4 sample injected at 5000 kPa, with a porosity of 8.09 mm^3^. These samples are close to the mean porosity. These samples are not included in Table 4 because they were not subjected to tensile tests.

### 3.3. Tensile Test Results 

**Figure 17:**
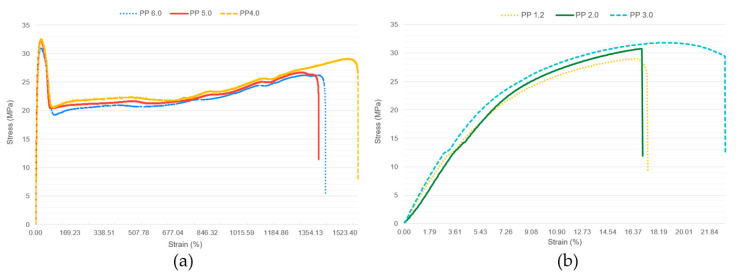
Tensile strength–strain curves. (**a**) Low injection temperature—Samples PP 4.0, PP 5.0, and PP 6.0. (**b**) High injection temperature—Samples PP 1.2, PP 2.0, and PP 3.0.

Crystalline polymers typically exhibit higher mechanical strength and dimensional stability. The regular packing of polymer chains in a crystalline lattice provides strong in-termolecular forces and enhances the load transfer between chains. Amorphous polymers, although generally less rigid, can exhibit greater flexibility and impact resistance due to their random chain arrangement. In the present work, the unreinforced PP samples have either amorphous or crystalline behavior depending on the process conditions. According to Carrasco et al. [38], the differential scanning calorimetry (DSC) of the virgin PP homopolymer at its melting and crystallization temperatures were 164.68 °C and 134.17 °C, respectively, and the material was 55.12% crystalline. From the numerical analysis of Table 4 data and Figure 17, it is observed that polypropylene behaves as a crystalline material when processed at higher temperatures and pressures. The strain values of samples PP 1.#, PP 2.#, and PP 3.#, below 35%, compared with the values for the samples PP 4.#, PP 5.#, and PP 6.#, above 1400%, provide evidence of the crystalline behavior of the first samples and the amorphous behavior of the latter. An interesting observation is also that all the reinforced samples behave like a crystalline polymer, where the strain reaches a maximum of 31.3%. 

Figure 17 provides information about the amorphous behavior of the PP under conditions of low injection temperature in case (a) and the crystalline behavior of the PP under conditions of high injection temperature in case (b). 

In order to analyze yield strength and strain with analogous scales, samples with elongations above 1000%, i.e., PP samples with amorphous behavior, PP 4.x, PP 5.x, and PP 6.x, were excluded. The amorphous samples were excluded to improve the graphical representation of the strain.

**Figure 18:**
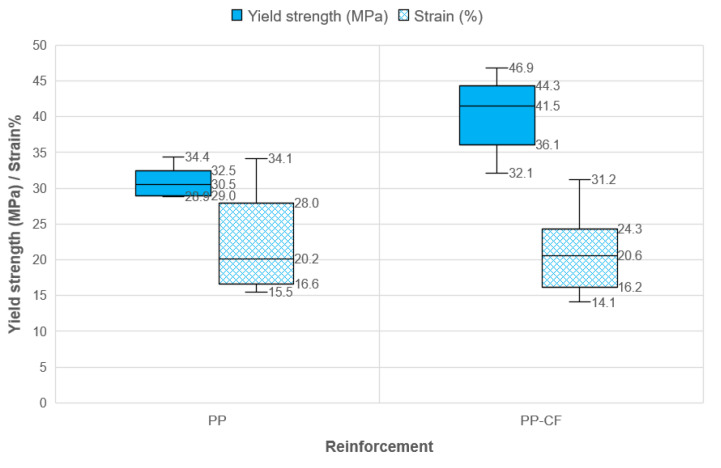
Comparison of yields strength and strain between PP samples without (PP) and with (PP-CF) carbon fiber reinforcement. (PP samples with amorphous behavior, PP 4.x, PP 5.x, and PP 6.x were excluded.).

Figure 18 represents the tensile behavior of the PP depending on whether or not it has reinforcement. An evaluation of this graph highlights that the yield strength increases by about 30%, while the strain remains practically unchanged. Adding brittle carbon fiber fabric reduces the elongation in PP-CF 4X, PP-CF 5.x, and PP-CF 6.x, but it is similar in PP 1x, PP2x, and PP3X with respect to PP-CF X.X. The results obtained for the unreinforced PP show a mean yield stress similar to those obtained by Yousef [32]. 

It is observed that the mean yield stress for PP is 30.8 MPa, and the mean yield with a value of 39.8 MPa is improved by 30% with the inclusion of the carbon fiber fabric reinforcement. However, in the mean strain, the material with reinforcement is maintained, but less variability is observed. 

**Figure 19:**
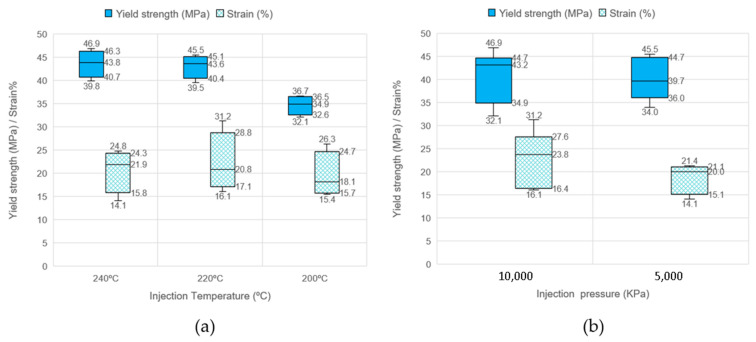
Comparison of the yield strength and strain at different injection parameters. (**a**) At different temperatures. (**b**) At different pressures.

Figure 19 presents the yield strength and strain versus the injection parameters, temperature and pressure, for the carbon-fiber-reinforced polypropylene. Looking at Figure 19a, comparing the yield strength at different temperatures, an interesting observation is that the mean yield strength is maximum for injection temperatures of 220 °C (43.6 MPa) and 240 °C (43.8 MPa) and represents an increase of 42% compared to the yield mean of non-reinforced material. However, in the case of an injection temperature at 200 °C, it is only 13% higher than in the case of the non-reinforced matrix. An anomalous behavior occurs in the case of a decrease at 200 °C of the deformation stress and a weak decrease in the elongation, which can be attributed to the high concentration of porosity in the PP-CF 5.2 (3.56%), PP-CF 6.0 (5.03%), and PP-CF 6.2 (4.86%) specimens. Similarly, Figure 19b shows the yield strength and strain versus the injection pressure. The highest yield strength result occurs at the highest pressure, but there is only an 8% increase.

**Figure 20:**
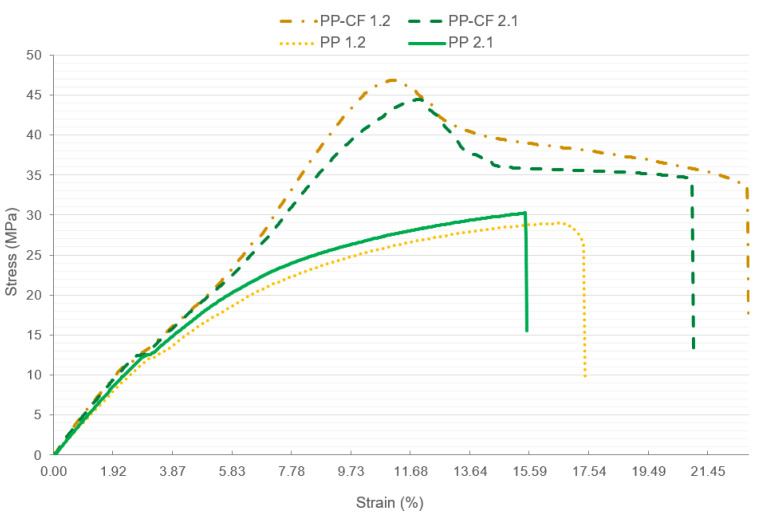
Comparison of the tensile strength–strain curves of samples PP 1.2, PP 2.1, PP-CF 1.2, and PP-CF 2.1.

Figure 20 shows the different stress–strain curves of the reinforced PP-CF 1.2 and PP-CF 2.1 samples compared to the non-reinforced samples PP 1.2 and PP 2.1. It is interesting to note that the most significant increases in yield stress came from the reinforced samples.

**Figure 21:**
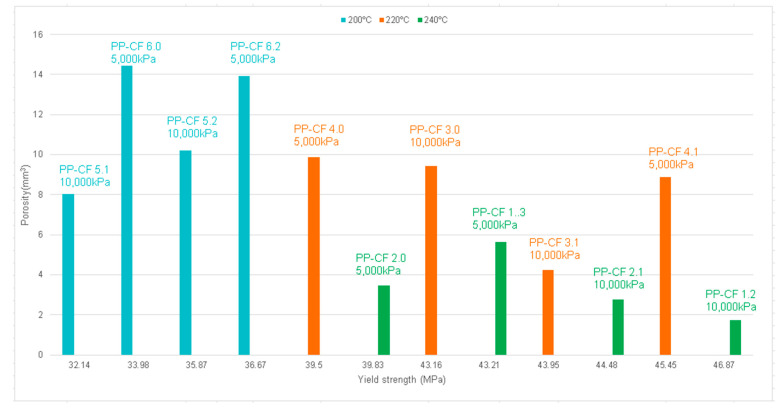
Porosity versus yield strength for carbon-fiber-reinforced polypropylene.

From the data presented in Figure 21, in which the relationship between porosity and yield strength is analyzed, it can be concluded that the lowest values of yield stress, with a porosity variability between 8 and 14 mm^3^, coincide with the reinforced samples injected at 200 °C. Likewise, it can also be observed that the reinforced samples with lower porosity, between 1.8 and 3.5 mm^3^, correspond to the specimens injected at 240 °C. In the cases of temperatures of 200 and 240 °C, it is assumed that the higher the temperature, the lower the porosity and the higher the yield stress. An anomalous behavior is observed in the specimens injected at a temperature of 220 °C, with significant variability in yield stress. 

Regarding the variability of porosity and its influence on the stress–strain curve, it should be noted that a study of pore diameter distribution has not been carried out, which should be analyzed in a future work. Differential scanning calorimetry tests should also be included in order to obtain the proportion of crystalline and amorphous material in the samples, which may clarify the anomalous behaviors in the tensile tests. 

## 4. Conclusions

The present study analyzes, from a mechanical and engineering process point of view, the polypropylene reinforced by means of injection molding with a carbon fiber fabric. The variables that were taken into account for the design of the experiments were the material (with or without carbon fiber fabric reinforcement), the injection temperature at three levels, and the injection pressure at two levels. These samples were geometrically analyzed through laser scanning, their internal defects were analyzed through computed tomography, and subsequently, their tensile mechanical properties were tested.

The following conclusions can be drawn from the experimental study:

- It can be stated that CF reinforcement has a positive influence on the shrinkage reduction of the PP material [36].
- The minimal shrinkage occurs with PP-CF at a temperature injection of 220 °C.
- The injection pressure has no influence on the decrease in shrinkage.
- The results of the CT scan of the samples without reinforcement have all come out with zero porosity, except for PP 3.2, which was excluded. The use of a holding pressure in the injection process inhibits the formation of internal porosity in the pure PP material [39].
- The tomographic results show that the carbon fiber fabric remains well-positioned in the central area.
- The porosity analysis denotes that with the PP-CF, the higher the temperature, the lower the porosity. With respect to pressure, porosity shows a slight inverse relationship with pressure.
- From the analysis of the results, it is observed that polypropylene behaves as a crystalline material when processed at higher temperatures and higher pressures.
- It is observed that the mean yield strength of PP-CF improves by 36% with the inclusion of carbon fiber fabric reinforcement
- The mean yield strength is highest for injection temperatures of 220 °C and 240 °C and represents an increase of 43% compared to the non-reinforced material.

Finally, further studies are needed on the effect of pressure and temperature on the polypropylene injection process as well as determining the crystallinity properties of the composite. Future studies to increase carbon fiber adhesion may also be relevant.

## Figures and Tables

**Table 1:** Polypropylene properties.

Properties	Value	Test Method
Melt Flow Rate (230 °C/2.16 kg)	12 g/10 min	ASTM D 1238 [21]
Density/Specific gravity	0.9 g/cm^3^	ASTM D 792 [22]
Flexural Modulus	1480 MPa	ASTM D 790 [23]
Tensile Stress at Yield	34 MPa	ASTM D 638 [24]
Tensile Elongation at Yield	10%	ASTM D 638

**Table 2:** Specifications of carbon fiber reinforcement.

Properties	Value
Diameter of fiber	5–8 μm
Density	1.76 g/cm^3^
Tensile strength	3950 MPa
Tensile modulus	238 GPa
Elongation at break	1.7%

**Table 3:** Design of experiments.

Experiment Number	Sample Number #	Sample Code	Carbon Fiber Fabric	Temperature (°C)	Pressure (kPa)
1	0 to 5	PP 1.#	No	240	10,000
2	0 to 5	PP-CF 1.#	Yes	240	10,000
3	0 to 5	PP 2.#	No	240	5000
4	0 to 5	PP-CF 2.#	Yes	240	5000
5	0 to 5	PP 3.#	No	220	10,000
6	0 to 5	PP-CF 3.#	Yes	220	10,000
7	0 to 5	PP 4.#	No	220	5000
8	0 to 5	PP-CF 4.#	Yes	220	5000
9	0 to 5	PP 5.#	No	200	10,000
10	0 to 5	PP-CF 5.#	Yes	200	10,000
11	0 to 5	PP 6.#	No	200	5000
12	0 to 5	PP-CF 6.#	Yes	200	5000

**Table 4:** Results of geometrical, tomographic, and tensile testing for the replicas selected.

Sample Code	Mean Width (mm)	SD Width (mm)	Mean Height (mm)	SD Height (mm)	Total Volume (mm^3^)	Pores (mm^3^)	Pores (%)	Yield Strength (MPa)	Strain (%)
PP 1.2	9.59	0.28	3.72	0.25	287.9	0	0	28.98	17.39
PP 1.3	9.59	0.27	3.60	0.32	288.0	0	0	28.88	25.93
PP 2.0	9.62	0.26	3.65	0.31	323.8	0	0.02	30.76	17.03
PP 2.1	9.66	0.25	3.62	0.35	323.9	0	0	30.25	15.49
PP 3.0	9.68	0.21	3.79	0.23	323.9	0	0	31.81	22.96
PP 3.1	9.75	0.20	3.75	0.27	324.0	0	0	34.38	34.14
PP 4.0	9.79	0.16	3.72	0.25	323.8	0	0	32.51	1588.90
PP 4.1	9.65	0.31	3.72	0.29	323.2	0	0	31.41	1387.44
PP 5.0	9.69	0.22	3.72	0.25	323.2	0	0	32.34	1394.73
PP 5.1	9.74	0.19	3.74	0.26	323.2	0	0	34.28	1454.73
PP 6.0	9.82	0.21	3.80	0.25	324.1	0	0	31.02	1428.15
PP 6.1	9.68	0.13	3.77	0.25	324.0	0	0	30.07	1396.76
PP-CF 1.2	9.88	0.21	3.90	0.16	285.7	1.74	0.61	46.87	22.76
PP-CF 1.3	9.88	0.24	3.89	0.21	282.1	5.65	1.96	43.21	24.79
PP-CF 2.0	9.84	0.21	3.91	0.19	294.9	3.45	1.16	39.83	14.08
PP-CF 2.1	9.87	0.15	3.89	0.17	296.0	2.75	0.92	44.48	20.96
PP-CF 3.0	9.91	0.16	3.95	0.20	314.0	9.42	2.91	43.16	16.09
PP-CF 3.1	9.99	0.17	3.92	0.19	319.7	4.24	1.31	43.95	31.24
PP-CF 4.0	9.99	0.30	3.97	0.17	277.6	9.88	3.44	39.50	21.35
PP-CF 4.1	9.84	0.13	3.95	0.19	278.7	8.88	3.09	45.45	20.28
PP-CF 5.1	9.86	0.13	3.99	0.45	276.9	8.03	2.82	32.14	26.33
PP-CF 5.2	9.87	0.23	3.93	0.26	276.7	10.22	3.56	35.87	16.45
PP-CF 6.0	9.88	0.23	3.95	0.22	272.7	14.44	5.03	33.98	15.44
PP-CF 6.2	9.85	0.21	4.00	0.22	273.2	13.94	4.86	36.67	19.81

## Data Availability

Data are contained within the article.
